# Prevalence of SARS-CoV-2 amongst ophthalmologists throughout the first and second waves of the pandemic

**DOI:** 10.1097/MD.0000000000028192

**Published:** 2021-12-17

**Authors:** Matteo Sacchi, Rosario Alfio Umberto Lizzio, Edoardo Villani, Elena Tagliabue, Gianluca Monsellato, Giorgio Pajardi, Saverio Luccarelli, Paolo Nucci

**Affiliations:** a Eye Clinic, San Giuseppe Hospital, IRCCS Multimedica, Milan, Italy; b Department of Clinical Science and Community Health, University of Milan, Milan, Italy; c Value-based Healthcare Unit, IRCCS Multimedica, Milan, Italy; d Plastic and Hand Surgery Clinic, San Giuseppe Hospital, IRCCS Multimedica, Milan, Italy.

**Keywords:** COVID-19, eye care professionals, infection, pandemic, SARS-CoV-2

## Abstract

The study aims to investigate the prevalence of severe acute respiratory syndrome coronavirus 2 (SARS-CoV-2) infection among ophthalmology unit staff throughout the first and second waves of the outbreak, in order to verify the effectiveness of the measures adopted in containing the contagion.

A retrospective observational study was conducted involving staff members, who received a naso/oropharyngeal swab when complaining of SARS-CoV-2 symptoms and once a month as a screening measure. They were tested for SARS-CoV-2 antibodies as a screening measure during the first and the second wave. Clinical activities performed during the outbreak were compared with those performed during the same period in 2019 and correlated with the number of coronavirus disease-2019 eye care workers.

Analysis included 25 workers. Clinical infection was 0% and 12% whereas the prevalence of SARS-CoV-2 antibodies ranged from 4% to 8% in the first and second wave, respectively. The increase in the prevalence of SARS-CoV-2 infection between the first and the second wave was not significant (1/25 vs 3/25, *P* = .6092). Clinical activities significantly decreased during the first wave compared with the same period in 2019 (3256 vs 10,075, *P* < .0001, –68% to 2019), but increased during the second wave (8208 vs 3256, *P* < .0001, +152% to the first wave).

Despite the increase in routine activities during the second wave, we did not observe a significant increase in SARS-CoV-2 prevalence. Strict protection measures seemed to contain the rate of contagion among the ophthalmology unit members even in a high-volume clinical setting in one of the most affected area by the coronavirus disease-2019 outbreak.

## 1 Introduction

On December 30, 2019 in Wuhan, China, Dr Li Wenliang, a fellow ophthalmologist, was the first to raise alarm about a possible outbreak of an illness similar to the severe acute respiratory syndrome that occurred in 2003, later recognized as coronavirus disease-2019 (COVID-19). He contracted the disease a few days later, and died a month later on February 7, 2020.^[1,2]^

The World Health Organization declared that the infection had spread worldwide on March 11, 2020.^[3]^ The first pandemic wave started in February 2020 and settled during the summer. From the beginning of the fall of 2020, Europe has been dealing with the second wave of the COVID-19 outbreak.

In 2021, 1 year after the onset of the COVID-19 outbreak, new severe acute respiratory syndrome coronavirus-2 (SARS-CoV-2) variants have been identified, with a significantly higher rate of transmission compared to the original strains. The B.1.1.7 variant, first identified in the UK, is rapidly spreading to other European countries and quickly increasing the number of new COVID-19 cases.^[4]^ Italy was the first European country to report the presence of COVID-19 disease and Lombardy, a region around Milan home to 10 million people, was the first and the most affected area in Italy during the first wave of the pandemic in 2020.^[5,6]^ In March 2021, COVID-19 cases are still rising in Italy due to the new SARS-CoV-2 variants, and we are likely getting close to a new pandemic of SARS-CoV-2. COVID-19 is a severe acute respiratory syndrome caused by a highly contagious RNA virus, SARS-CoV-2. Among medical activities, ophthalmologists’ practice is of special concern. First, it is worth noting that although fever and respiratory symptoms are the most common features of the disease, non-respiratory features have been reported, including ocular manifestations.^[7,8]^

Keratoconjunctivitis can be the initial presentation of COVID-19, and the involvement of the conjunctiva has been reported in up to 31.6% of COVID-19 patients.^[7,9–12]^ As the mean duration of conjunctival congestion has been reported to be a few days, ocular involvement is likely to be underestimated.^[11]^

It is worth noting that patients with undiagnosed COVID-19 in the prodromal stage may be referred to an ophthalmologist if they complain of ocular problems; this puts ophthalmologists and professional eye care staff at risk of infection.^[10,13]^ Asymptomatic patients who undergo routine eye examinations can also spread the infection, as it has been shown that asymptomatic carriers of SARS-CoV-2 can transmit the disease.^[14]^ The ocular route is a well-known path of transmission for respiratory viruses and it has been suggested to be an effective path for SARS-CoV-2 as well.^[8,15,16]^

Eye visits require close proximity between the ophthalmologist and patient and include possible contact between the doctor's fingers and the patient's conjunctival mucosal surfaces. In addition, as pointed out in the review by Minas Theodore Coroneo, during slit-lamp biomicroscopy, the overlapping between the breathing zones of the physician and the patient is likely to promote the airborne transmission of respiratory droplets, thus favoring contagion.^[1]^

As COVID-19 spreads through person-to-person transmission, personal contact is of great relevance to the spread of infection. Hospitals were first considered places of paramount importance in outbreak control because of the high flow of incoming patients. During the outbreak, our ophthalmology unit staff, together with other units of our hospitals, adopted measures for the control of the infection, including the reduction of the flow of outpatients inside the hospital, the use of personal protection equipment (PPE), and the temporary cessation of non-urgent activities.

In order to verify the effectiveness of the measures we adopted in our hospital and among the ophthalmology unit members in containing the SARS-CoV-2 infection, we investigated the prevalence of SARS-CoV-2 infection, including symptoms, nasopharyngeal/oropharyngeal swab data, and the presence of antibodies against SARS-CoV-2 among our ophthalmology unit staff, throughout the first and second waves of the COVID-19 outbreak, also taking into account the volume of clinical activity.

## 2 Methods

In this retrospective analysis, we included staff of the Ophthalmic Unit (ophthalmologists, residents, and orthoptists) who completed the questionnaire (see below) and who underwent a nasopharyngeal/oropharyngeal swab and blood test for the presence of SARS-CoV-2 antibodies during the first and second waves who were willing to participate in the study. We considered the period between March 2020 and May 2020 as the ‘first wave’ and between September 2020 and November 2020 as the ‘second wave’. We used a questionnaire to collect the following demographic and clinical data: the onset of clinical features related to SARS-CoV-2 disease from the beginning of the outbreak, age at the time of the test, sex, coexisting diseases, and previous diagnosis of COVID-19. The staff members were also asked about PPE (daily use of new PPE, uncommon reuse of PPE, common reuse of PPE), and contact with suspected or confirmed COVID-19 patients.

A nasopharyngeal/oropharyngeal swab was performed on each subject who complained of SARS-CoV-2 related symptoms and once a month as a screening measure during the first and second waves of the outbreak. The eye care staff of the Ophthalmic Unit of San Giuseppe Hospital were tested for SARS-CoV-2 immunoglobulin M (IgM) and immunoglobulin G (IgG) antibodies as a screening measure at 2 months from the beginning of the outbreak in Italy (first wave) and at 2 months after the onset of the second wave. Tests for SARS-CoV-2 antibodies were also performed for each subject with a positive nasopharyngeal/oropharyngeal swab.

In addition, we retrospectively analyzed electronic medical records and collected the number of eye examinations performed during both the first and second waves of the outbreak (March–May 2020 and September–November 2020). The number of clinical activities performed during the outbreak was compared with those performed during the same period in 2019 (March–May 2019 and September–November 2019). We correlated the number of COVID-19 eye care workers with the number of eye examinations performed.

As the plastic and hand surgery unit staff were also tested for the presence of SARS-CoV-2 antibodies as a screening measure, we considered the SARS-CoV-2 seroprevalence among the Plastic and Hand Surgery Unit of our Hospital during both the first and second waves of the outbreak as a control group of physicians not exposed to the specific eye-care-related risks of infection.

The study and data collection were carried out with approval from the local Institutional Review Board (IRCCS Multimedica) and adhered to the tenets of the Declaration of Helsinki. Written informed consent was obtained from all subjects who participated in the study.

### 2.1 Test technique

#### 2.1.1 Serological test

After collection, venous blood samples were stored at −20°C until use. Serum was tested for the qualitative presence of SARS-CoV-2 IgM and IgG antibodies using an immunochromatographic lateral flow assay (KHR25COR, Technogenetics Srl). In summary, 10 μL of serum or whole blood was placed in the sample port, followed by the addition of 2 to 3 drops (70–100 μL) of dilution buffer. After 15 minutes of incubation at room temperature, the results were visually recorded as lines in the control zone (internal control) and test zone (IgM and IgG antibodies). If the sample was positive, the subject was tested using 2 confirmatory swabs to assess the actual positivity.^[17]^

#### 2.1.2 Nasopharyngeal/oropharyngeal swabs

The swabs were performed by well-trained healthcare workers and maintained between 2°C and 8°C. All samples were analyzed using the Abbott RealTime SARS-CoV-2 assay, a qualitative real-time assay performed on the Abbott m2000 platform. This assay amplifies the target regions of the SARS-CoV-2 RNA-dependent RNA polymerase (RdRp) gene and the N gene with the same fluorophore within a single well. Each run also included 2 controls, 1 positive and 1 negative, provided by the manufacturer. The m2000rt system then interpreted the amplification curves and reported the results as detected or not detected.^[18]^

### 2.2 Statistical analysis

Continuous variables are expressed as the mean (±standard deviation) and categorical variables are reported as frequencies and percentages. SARS-CoV-2 positivity was defined as IgG, IgM, and nasopharyngeal/oropharyngeal swabs that were positive for SARS-CoV-2 in the first or second wave of the outbreak. Odds ratios (ORs) and 95% confidence intervals (CIs) were calculated using bivariable logistic regression models to assess whether age or employment characteristics were associated with SARS-CoV-2 positivity. The chi-square test or Fisher exact test was used to evaluate differences between the frequency of SARS-CoV-2 positivity amongst team members and differences in the number of ambulatory visits performed. Statistical analyses were performed using SAS software version 9.4 (SAS Institute Inc., Cary) and *P* values < .05 were considered statistically significant.

## 3 Results

A total of 25 members of the Ophthalmology Unit of San Giuseppe Hospital (13 consultants, 7 residents, 5 orthoptists) who completed the questionnaire and underwent a nasopharyngeal or oropharyngeal swab and a qualitative serological test for SARS-CoV-2 antibodies during the first and second waves were included in the analysis. All staff members were included in the analysis. The baseline demographic and clinical features of the subjects are summarized in Table 1. The subjects’ median age was 39 years (min–max, 24–61), and 64% were male (16/25). Three out of 25 subjects (12%) had previous comorbidities (hypertension and asthma).

**Table 1:** Population characteristics.

	First wave	Second wave
Subjects	N = 25	
M/F	16/25 (64%)	
Age (median, min–max)	39 (24–61)	
Previous comorbidity	3 (12%)	
Symptoms	0 (0%)	3 (12%)
IgM positive	1 (4%)	2 (8%)
IgG positive	0 (0%)	2 (8%)
Positive swab	0 (0%)	3 (12%)
SARS-CoV-2 positivity in the other OU^∗^	6%	12%
Daily use of new PPE	23 (92%)	24 (96%)
Uncommon reuse of PPE	2 (8%)	1 (4%)
Contact with COVID-19 patients	2 (8%)	4 (16%)

Data are presented as frequency and percentage unless otherwise noted. COVID-19 = coronavirus disease-2019, F = female, IgG = immunoglobulin G, IgM = immunoglobulin M, M = male, N = number, OU = operative unit, PPE = personal protection equipment, SARS-CoV-2 = severe acute respiratory syndrome coronavirus-2.

∗ Indicates the Plastic and Hand Surgery Unit.

### 3.1 First wave

#### 3.1.1 Symptoms

None of the participants reported COVID-19-compatible symptoms or a previously confirmed diagnosis of COVID-19.

#### 3.1.2 Real time-polymerase chain reaction analysis and seroprevalence

The 25 members of the team were tested via a nasopharyngeal/oropharyngeal swab on June 8, 2020 as a screening measure. None of the tests revealed the presence of viral RNA. Among the included subjects, 1 tested positive for IgM antibodies (4%) and none tested positive for IgG antibodies (Table 1). A worker with IgM antibodies against SARS-CoV-2 was placed in quarantine. The nasopharyngeal/oropharyngeal swab performed 2 days and 1 month later was negative, and until October 2020, IgG against SARS-CoV-2 was undetected.

#### 3.1.3 Eye examination

Throughout the period between March and May 2020, the total number of eye examinations significantly decreased compared with the same period in 2019 (3256 vs 10,075, *P* < .0001, chi-square test, –68% to 2019).

#### 3.1.4 SARS-CoV-2 prevalence amongst Plastic and Hand Surgery staff (control group)

Among the 33 members, including 13 consultants, 6 residents, and 14 therapists, 2 consultants (6%) experienced fever, fatigue, and anosmia. Nasopharyngeal/oropharyngeal swabs confirmed the presence of viral RNA. Blood tests revealed IgM and IgG antibodies against SARS-CoV-2. Both recovered fully after 21 days of quarantine. None of the other team members tested positive for viral RNA or SARS-CoV-2 antibodies during the first wave.

### 3.2 Second wave

#### 3.2.1 Symptoms

As of November 2020, 2 residents and 1 consultant (3/25 members, 12% of the staff) experienced COVID-19 related symptoms: 3 experienced fever, 2 had a sore throat, 2 had anosmia, and 2 had fatigue.

#### 3.2.2 Real time-polymerase chain reaction analysis and seroprevalence

All patients were positive for nasopharyngeal/oropharyngeal swabs, and the 2 residents (8% of the staff) had IgM and IgG antibodies against SARS-CoV-2; the consultant did not have SARS-CoV-2 antibodies. None of the patients reported any comorbidities and none required hospitalization. After quarantining for 14 days (1 resident and 1 consultant) and 21 days (1 resident), the nasopharyngeal/oropharyngeal swab was negative, and the subjects had fully recovered. As of November 2020, none of the other team members showed IgM or IgG antibodies against SARS-CoV-2, and the nasopharyngeal/oropharyngeal swabs showed negative real time-polymerase chain reaction (real time-PCR) results.

#### 3.2.3 Eye examination

Throughout the period between September and November 2020, we observed a significant increase in the total number of eye examinations compared to the first wave (8208 vs 3256, *P* < .0001, chi-square test, +152% compared with the first wave) despite a significant reduction compared to the same period in 2019 (8208 vs 10,252, *P* < .0001, 20%–20% to 2019).

#### 3.2.4 Prevalence of SARS-CoV-2 amongst Plastic and Hand Surgery staff (control group)

Four out of 33 team members (12%) experienced COVID-19 related symptoms (2 reported fever, sore throat, and anosmia, and 2 reported fever and anosmia). They were positive for both nasopharyngeal and oropharyngeal swabs and IgM and IgG antibodies against SARS-CoV-2. They fully recovered after 15 days (2 members) and 21 days (2 members) of quarantine. None of the other team members tested positive for nasopharyngeal or oropharyngeal swabs or SARS-CoV-2 antibodies during the second wave of the outbreak.

### 3.3 COVID-19 infection rate and clinical activity volume

Despite a significant increase in the total number of eye examinations during the second wave of the outbreak compared to the first wave, we did not observe a significant increase in the prevalence of SARS-CoV-2 infection (1/25 vs 3/25, *P* = .6092, Fisher exact test). Considering the first and second wave of the outbreak together, the number of cases in the Ophthalmology Unit did not statistically differ from those of the Plastic and Hand Surgery Units (4/25 vs 6/33, *P* = 1.00). Logistic models to evaluate the association between SARS-CoV-2 positivity and the collected variables did not show statistically significant results, however, we found an interesting trend in the ORs for the analyzed Ophthalmology Unit (Table 2).

**Table 2:** Association between collected variables and SARS-CoV-2 positivity.

	Overall		Second wave	
	OR (95%CI)	*P* value	OR (95%CI)	*P* value
Model 1: age	1.06 (0.94–1.19)^∗^	0.3617	1.05 (0.91–1.20)^∗^	0.4987
Model 2: age <39 years (vs ≥39 years)	3.57 (0.35–6.93)	0.2856	2.40 (0.19–30.52)	0.4998
Model 3: resident (vs not resident)	2.83 (0.36–22.40)	0.3235	6.80 (0.50–91.49)	0.1483

CI = confidence interval, OR = odds ratio, SARS-CoV-2 = severe acute respiratory syndrome coronavirus-2.

∗ OR for a 1-unit decrease.

We found an overall 6% increase in the risk of positivity for each year of age decrease (OR; 95%CI, 1.06; 0.94–1.19). The same results were obtained considering only the second wave of the outbreak (OR; 95%CI, 1.05; 0.91–1.20). Considering the median age as the cut-off, we found that being younger than 39 years was more strongly associated with SARS-CoV-2 positivity, either overall or only in the second wave (ORs 3.57 and 2.40, respectively). Finally, residents showed an increased risk of SARS-CoV-2 positivity compared to non-residents (OR = 2.83 overall and OR = 6.80 for second wave).

## 4 Discussion

Here, we report the prevalence of SARS-CoV-2 infection amongst our team of eye care providers working in Italy, the first European country affected by the COVID-19 outbreak. During the first wave, no members on the team experienced COVID-19 symptoms. All team members underwent serological testing 2 months from the beginning of the COVID-19 outbreak as a screening measure; 1 asymptomatic subject (4%) was found to have IgM antibodies against SARS-CoV-2. Two days later, the nasopharyngeal/oropharyngeal swab was negative for active infection. To date, IgG antibodies have not been detected against SARS-CoV-2 in the patient. Since we used a qualitative test with low sensitivity to detect the presence of SARS-CoV-2 antibodies, and because of the negative result of the nasopharyngeal/oropharyngeal swab, we can assume that the test result was a false positive.^[19]^ It should also be noted, however, that in a small proportion of COVID-19 patients, only 1 antibody isotype could be detected.^[20]^

During the second wave, 3 team members experienced COVID-19 symptoms (12%). They had a mild form of the disease and fully recovered after a quarantine period. They tested positive for COVID-19 on real time-PCR analysis from nasopharyngeal/oropharyngeal swabs; however, only 2 out of 3 COVID-19 positive subjects also showed SARS-CoV-2 antibodies (8%).

In our analysis, we included a control group, that is, the Plastic and Hand Surgery Unit staff, as they do not share with ophthalmologists the related risk of contagion due to eye examination. The SARS-CoV-2 prevalence between the Ophthalmology and Plastic and Hand Surgery Unit staff of the same hospital throughout the first and second wave was comparable (4%–8% vs 6%–12%, respectively; *P* value = 1.00. for both comparisons). We can conclude that despite the specific risk related to ophthalmic activities, the protective measures we adopted were able to maintain the risk of infection close to the prevalence observed in another unit of the same hospital.

Healthcare workers are considered to be at high risk of contagion, with a risk of infection up to 12-fold greater than that in the general community.^[21]^ In New York, the SARS-CoV-2 seroprevalence of healthcare workers has been reported to be up to 36%^[22]^ and studies carried out in Europe reported a SARS-CoV-2 seroprevalence ranging from 1% to 11%.^[20,23–28]^ These studies referred to health care workers, including clinical and non-clinical staff, both with and without COVID-19 patient exposure. Although considered a category at risk of infection,^[13,29]^ to the best of our knowledge, few data are available on SARS-CoV-2 seroprevalence among ophthalmologists and eye care workers.

The ocular route for the transmission of viral infection has been known from the beginning of the 1900s, and this should not be surprising, as the eye and lacrimal-nasal pathway seem to be an efficient route to enter the upper respiratory tract.^[15]^ Although ocular involvement is relatively rare after coronavirus infection, respiratory viruses have been shown to have ocular tropism.^[16]^ Since the first SARS-CoV outbreak in 2003, it has been known that the virus can be detected in the tear fluid, can be transmitted through mucous membranes, including ocular mucous membranes (conjunctiva), and can cause a respiratory infection following ocular involvement.^[16,30,31]^

The role of the ocular route for SARS-CoV-2 transmission was first noted by Dr Guangfa Wang, a respiratory specialist who developed a SARS-CoV-2 infection after his inspection at the Wuhan Fever Clinic. He wore an N95 mask, but did not have any eye protection during the site visit. As he experienced ocular involvement, including redness and conjunctivitis a few days before the onset of pneumonia, he concluded that the eyes were likely to be a route of infection.^[8]^ The observation of Dr Wang corroborates the protective role of eyeglasses against virus transmission among healthcare workers.^[32]^ Similar to SARS-CoV, SARS-CoV-2 has also been detected in tear film and conjunctival swab specimens of patients with confirmed COVID-19 infection.^[12,30,33,34]^ In addition, the cellular entry of SARS-CoV-2 depends on the angiotensin-converting enzyme 2 receptor, and the presence of the angiotensin-converting enzyme 2 receptor has been confirmed in the limbus, corneal epithelium, and superficial conjunctival cells.^[35]^

In a multicenter study conducted in Wuhan, China during the first wave of COVID-19, the incidence of SARS-CoV-2 antibodies among eye professionals was 2.52%. The authors concluded that ophthalmologists and eye care professionals have a similar risk of contagion to other healthcare workers.^[36]^

In our study, we reported the presence of SARS-CoV-2 antibodies in 4% and 8% of Ophthalmic Unit staff throughout the first and second waves of the COVID-19 outbreak, respectively. The presence of SARS-CoV-2 antibodies in our staff during the first wave seems to be comparable to the seroprevalence among European health care workers (1%–11%) and eye care professional staff in Wuhan, China (2.52%).^[20,23–27]^ The disease course was more severe in the study carried out in Wuhan than in our study. The higher rates of contact with COVID-19 patients (84%) and lack of PPE (54%) compared to our study (first wave: 8% and 8%, respectively; second wave: 4% and 16%, respectively) can at least partially explain the difference in disease severity between the 2 studies. Our study and other studies have found that being a young worker is a risk factor for the development of COVID-19.^[20,26,28]^ Junior staff workers are likely to be less trained and experienced with personal protective protocols. This could partially explain why being a younger worker may be associated with a higher risk of contracting COVID-19.

As COVID-19 contagion has been confirmed to spread through person-to-person transmission,^[37]^ the role of personal contact and proximity is considered to be of great relevance in outbreak control. In addition, SARS-CoV-2 has been shown to have a long incubation time,^[38]^ and more than half of patients do not have a fever at presentation.^[7]^ This means that afebrile patients in the early stage of the disease can be missed by surveillance protocols based on fever detection, thus increasing the spread of contagion.

In light of this, the reduction in the flow of outpatients inside the hospital was one of the first measures that was implemented, together with the use of PPE, to handle the outbreak. Since the outbreak in Italy in March, measures to contain the spread of infection have been adopted in our hospital, similar to those adopted in other hospitals in our country.^[13]^ Briefly, patients are only allowed to enter the hospital if their body temperature is below 37.5°C. Further, before entering the hospital, all patients must give the following written statement: ‘I declare to be healthy, with no signs or symptoms related to SARS-CoV-2, and I am not in quarantine for any previous SARS-CoV-2 infection’.

Ophthalmologists are considered to be at high risk of contagion because of the ocular tropism of the virus, the high volume of clinics, and the peculiarity of eye examinations.^[1,13,36]^ As a measure of protection, the eye care staff wore a filtering facepiece-2. It is worth noting that the filtering facepiece-2 masks have to be worn by the staff not only during the eye examination, but throughout the full working day, including in the locker room, restroom, common areas, and when moving inside the hospital. Eye-protective goggles were available and recommended when dealing with patients with conjunctivitis, and they were mandatory when visiting hospitalized COVID-19 patients. Slit lamps were equipped with breath shields, and ophthalmic equipment was disinfected with an alcohol-based solution between visits. It was recommended that eye care providers use an alcohol-based gel to clean their hands frequently and always between visits. The time between visits was increased up to 30 minutes to minimize person-to-person contact. The capacities of the waiting areas were reduced to one-third compared with the standard capability, with a distance of 2 m between seated patients. Routine, deferrable eye examinations and non-urgent surgical activities have been cancelled and rescheduled. Altogether, these measures significantly decreased the patient flow inside the hospital.

During the first wave, the number of routine visits decreased by 68% compared to the same period in 2019. Interestingly, despite a significant increase in clinical activities during the second wave (eye examinations +152% compared to the first wave), we did not observe a statistically significant higher number of staff who contracted COVID-19 during the second wave (1/25 vs 3/25, *P* = .6092). This means that PPE and infection control measures were also effective during the second wave, when clinical activities increased, as the number of COVID-19-positive workers was statistically comparable with those observed during the first wave.

Among the limitations of our study, we could not establish whether the infections were community or hospital-acquired. This study was carried out considering data from an eye care professional team working in a hospital in Milan, Italy, one of the countries hit worst by the COVID-19 outbreak. Further, the sample size was relatively small (25 subjects), the mean age was under 40 years, and members had a low rate of comorbidities. Therefore, we are aware that our results cannot be generalized and should be interpreted with caution when applied to subjects with different characteristics and operating in different working environments. As the rate of COVID-19 cases amongst our staff was low, we are aware that we cannot draw any strong conclusions from our logistic models, and we are also aware that cases are too low in our analysis to strongly conclude that being younger than 39 years and being a resident are risk factors for contagion.

Among the strengths of our study, this is, to the best of our knowledge, the first study on the prevalence of SARS-CoV-2 infection among eye care workers in Europe. We also believe that it is the first study to report SARS-CoV-2 related symptoms, real time-PCR analysis of nasopharyngeal/oropharyngeal swabs, and seroprevalence for the same team throughout the first and second COVID-19 outbreak waves. Lastly, we introduced a control group and were able to compare our eye care team's prevalence with the prevalence of other healthcare workers in the same hospital.

In conclusion, ophthalmologists and eye care providers play crucial roles in the COVID-19 outbreak.^[1]^ This is true for several reasons: ocular involvement can precede systemic manifestations and is associated with more severe systemic disease, the ocular route is a known path for virus contagion, and eye examinations using slit-lamp biomicroscopy can promote the transfer of respiratory droplets between physicians and patients.^[1,9–12]^

Our study showed that eye care professionals are at risk of contagion, however, we found that by decreasing the flow of patients and adopting strict protection measures, the rate of contagion among the members of a team of professional eye care staff can be significantly contained, even when working in an area that is one of the most affected by the COVID-19 outbreak. We believe that a better understanding of the risk of contagion among eye care staff, together with the protective measure we adopted to contain this risk, could be helpful, especially at present, as we are getting close to a new pandemic wave. We hope that our study will encourage further research on the rate of COVID-19 contagion and the most effective protective measure among those belonging to the professional eye care community.

## Author contributions

All authors contributed to the study design; M.S., R.A.U.L., G.M., S.L., performed the experimental part (data collection); all authors contributed to the data analysis;

G.M., R.A.U.L., contributed to the tables editing; M.S., R.A.U.L., G.M., E.V., E.T., contributed to the first draft writing; all authors read the manuscript and contributed to its final version.

**Conceptualization:** Matteo Sacchi, Rosario Alfio Umberto Lizzio, Gianluca Monsellato.

**Data curation:** Matteo Sacchi, Rosario Alfio Umberto Lizzio, Elena Tagliabue.

**Formal analysis:** Elena Tagliabue.

**Methodology:** Edoardo Villani.

**Supervision:** Matteo Sacchi, Edoardo Villani, Giorgio Pajardi, Saverio Luccarelli, Paolo Nucci.

**Validation:** Matteo Sacchi, Edoardo Villani, Giorgio Pajardi, Saverio Luccarelli, Paolo Nucci.

**Visualization:** Matteo Sacchi.

**Writing – original draft:** Matteo Sacchi, Rosario Alfio Umberto Lizzio, Edoardo Villani, Gianluca Monsellato.

**Writing – review & editing:** Matteo Sacchi, Edoardo Villani, Gianluca Monsellato, Paolo Nucci.
